# Supplementary figures and images for: Mathematical Modeling of the Evolution of Absenteeism in a University Hospital over 12 Years
Source: Int J Environ Res Public Health. 2022 Jul 6;19(14):8236. doi: 10.3390/ijerph19148236 (PMC9316583; doi:10.3390/ijerph19148236)

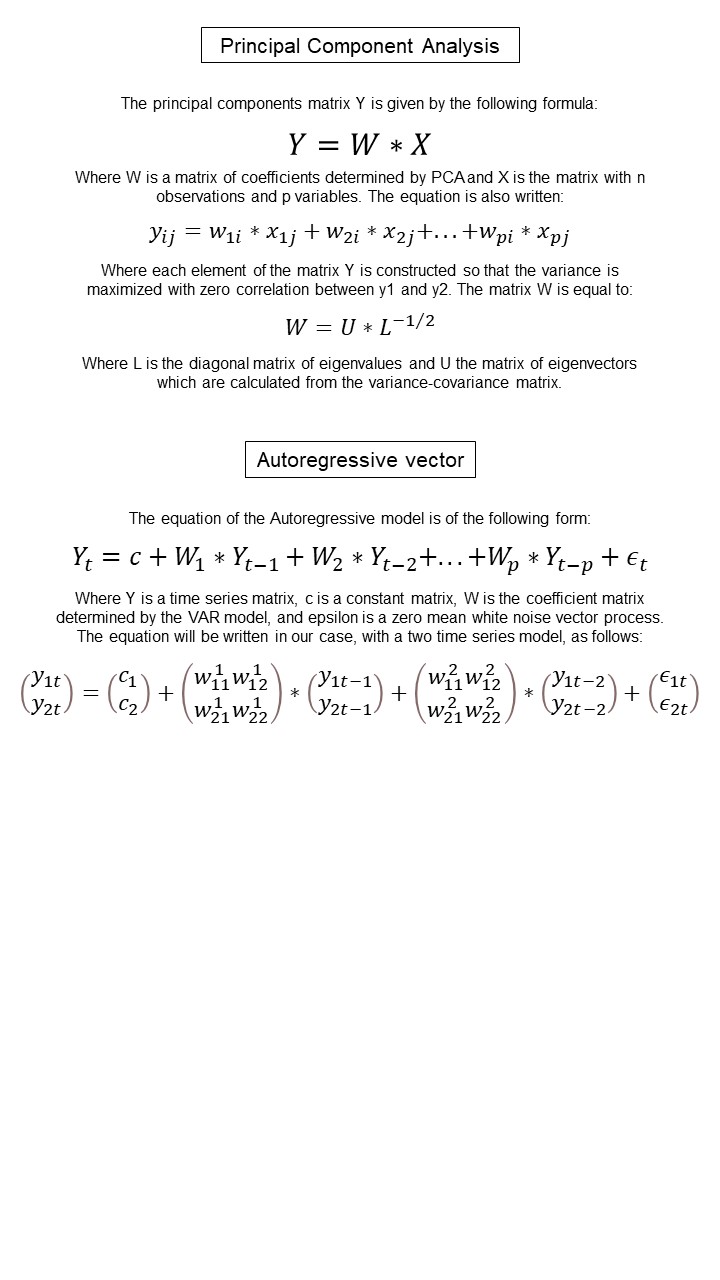

Supplement: Supplementary file 1 [file ijerph-19-08236-s001.zip › Equation (S1).jpg]

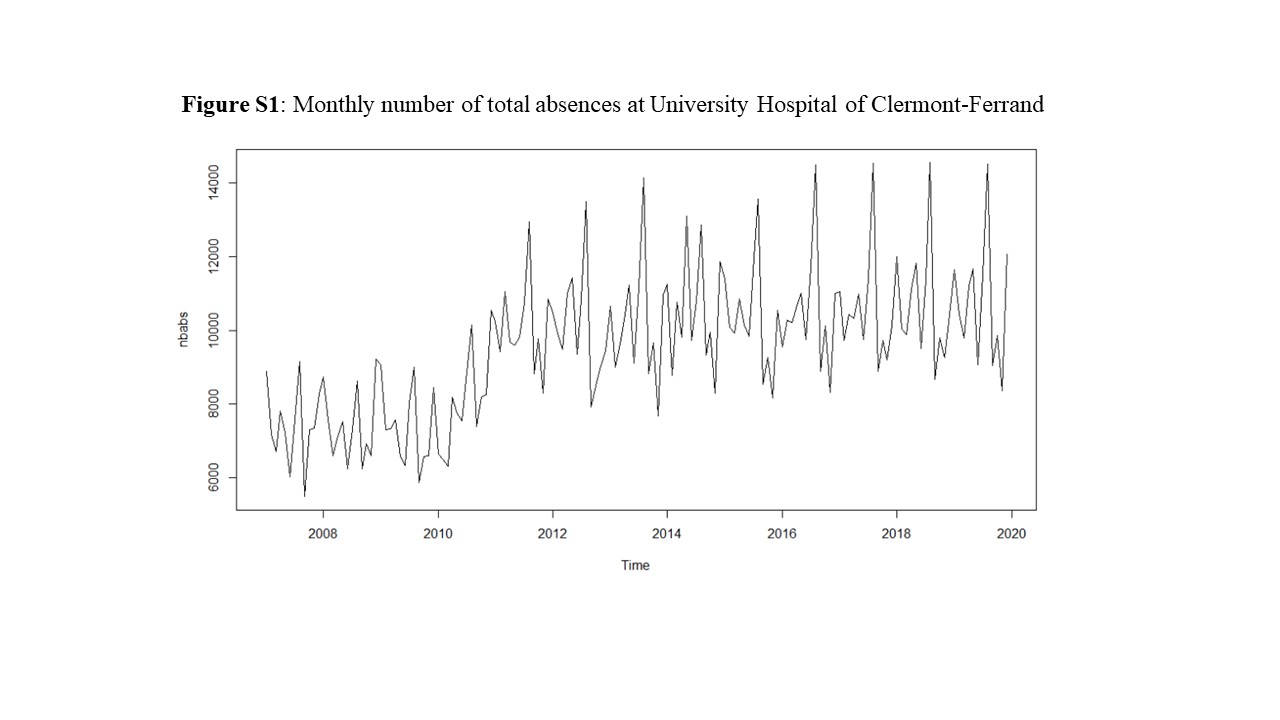

Supplement: Supplementary file 1 [file ijerph-19-08236-s001.zip › Figure S1.jpg]

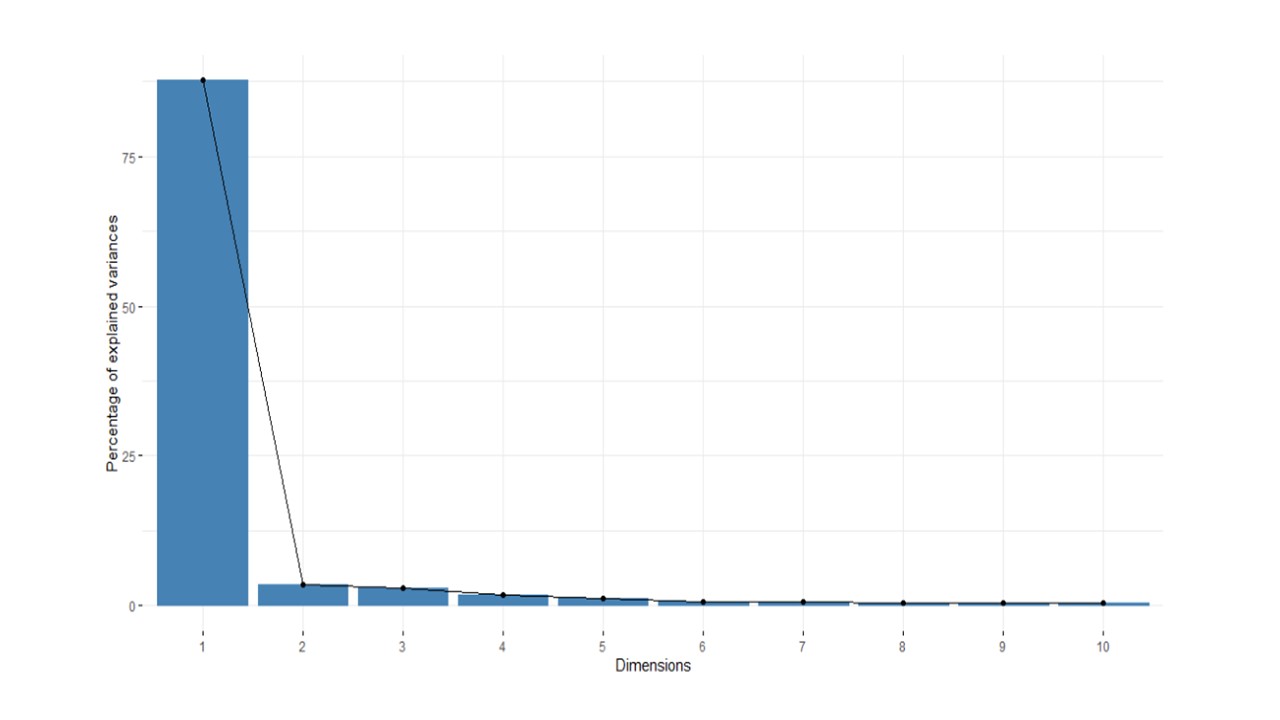

Supplement: Supplementary file 1 [file ijerph-19-08236-s001.zip › Figure S2.jpg]

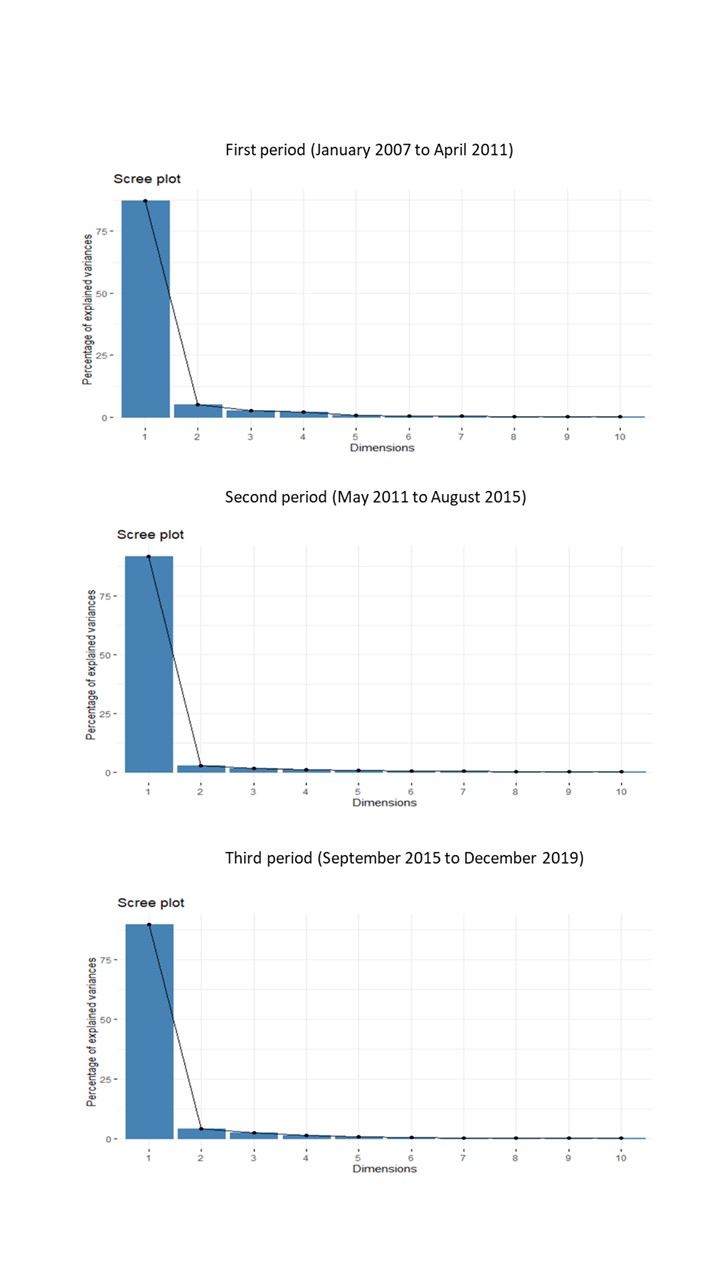

Supplement: Supplementary file 1 [file ijerph-19-08236-s001.zip › Figure S3.jpg]
